# Delay of airway epithelial wound repair in COPD is associated with airflow obstruction severity

**DOI:** 10.1186/s12931-014-0151-9

**Published:** 2014-11-27

**Authors:** Jeanne-Marie Perotin, Damien Adam, Juliette Vella-Boucaud, Gonzague Delepine, Sebastian Sandu, Anne-Carole Jonvel, Alain Prevost, Gérard Berthiot, Christophe Pison, François Lebargy, Philippe Birembaut, Christelle Coraux, Gaëtan Deslee

**Affiliations:** Department of Respiratory Diseases, University Hospital, 45 rue Cognacq Jay, 51100 Reims, France; INSERM UMRS 903, University Hospital, Reims, France; Department of Cardio-Thoracic Surgery, University Hospital, Reims, France; Department of Respiratory Medicine, Hospital, Charlevilles-Mezières, France; Institut Jean Godinot, Reims, France; Department of Respiratory Medicine, Hospital, Chalons en Champagne, France; Clinique Universitaire de Pneumologie, Pôle Thorax et Vaisseaux, CHU Grenoble; Inserm1055; Université Joseph Fourier, Grenoble, France; Department of anatomopathology, Pol Bouin Laboratory, University Hospital, Reims, France

**Keywords:** COPD, Airway epithelium, Bronchial cells, Bronchiolar cells, Wound repair, Cell proliferation

## Abstract

**Background:**

Airway epithelium integrity is essential to maintain its role of mechanical and functional barrier. Recurrent epithelial injuries require a complex mechanism of repair to restore its integrity. In chronic obstructive pulmonary disease (COPD), an abnormal airway epithelial repair may participate in airway remodeling. The objective was to determine if airway epithelial wound repair of airway epithelium is abnormal in COPD.

**Methods:**

Patients scheduled for lung resection were prospectively recruited. Demographic, clinical data and pulmonary function tests results were recorded. Emphysema was visually scored and histological remodeling features were noted. Primary bronchial epithelial cells (BEC) were extracted and cultured for wound closure assay. We determined the mean speed of wound closure (MSWC) and cell proliferation index, matrix metalloprotease (MMP)-2, MMP-9 and cytokines levels in supernatants of BEC 18 hours after cell wounding. In a subset of patients, bronchiolar epithelial cells were also cultured for wound closure assay for MSWC analyze.

**Results:**

13 COPD and 7 non COPD patients were included. The severity of airflow obstruction and the severity of emphysema were associated with a lower MSWC in BEC (p = 0.01, 95% CI [0.15-0.80]; p = 0.04, 95% CI [−0.77;-0.03] respectively). Cell proliferation index was decreased in COPD patients (19 ± 6% in COPD vs 27 ± 3% in non COPD, p = 0.04). The severity of COPD was associated with a lower level of MMP-2 (7.8 ± 2 10^5^ AU in COPD GOLD D vs 12.8 ± 0.13 10^5^ AU in COPD GOLD A, p = 0.04) and a lower level of IL-4 (p = 0.03, 95% CI [0.09;0.87]). Moreover, higher levels of IL-4 and IL-2 were associated with a higher MSWC (p = 0.01, 95% CI [0.17;0.89] and p = 0.02, 95% CI [0.09;0.87] respectively). Clinical characteristics and smoking history were not associated with MSWC, cell proliferation index or MMP and cytokines levels. Finally, we showed an association of the MSWC of bronchial and corresponding bronchiolar epithelial cells obtained from the same patients (p = 0.02, 95% CI [0.12;0.89]).

**Conclusion:**

Our results showed an abnormal bronchial epithelial wound closure process in severe COPD. Further studies are needed to elucidate the contribution and the regulation of this mechanism in the complex pathophysiology of COPD.

**Electronic supplementary material:**

The online version of this article (doi:10.1186/s12931-014-0151-9) contains supplementary material, which is available to authorized users.

## Background

Chronic obstructive pulmonary disease (COPD) is a heterogeneous disease characterized by progressive airflow limitation, variously associating airway inflammation and remodeling, lung parenchymal destruction, systemic inflammation and comorbidities as cardiovascular disease and metabolic syndrome [1,2]. COPD represents a major worldwide health topic in its morbidity, mortality and its large consumption of health care resources, with a limited effect of current treatments [3]. The development of new therapeutic strategies requires a better understanding of the mechanisms underlying the setting and/or progression of COPD.

Airway epithelium plays a major role of structural and functional barrier, preventing inhaled substances from entering the airway tissue, and coordinating the activities of cells of the innate and adaptive immune system. Exposure to tobacco smoke, allergens, airborne particulates, infectious agents and noxious gases can induce injuries of airway epithelium, requiring a regulated repair mechanism to restore its functionality [4].

After injury, epithelial cells initiate several repair process, including spreading and migration of cells neighboring the wound, proliferation, and progressive re-differentiation until a complete regeneration of a pseudostratified mucociliary epithelium [4]. Remodeling of large and small airways have been shown in COPD, including squamous metaplasia, airway wall fibrosis, goblet cell hyperplasia, submucosal gland hypertrophy, increase in bronchial smooth muscle [5]. Chronic and acute injuries by tobacco smoke, environmental irritants, microorganisms are thought to participate to epithelial remodeling [6]. However, whether there is an abnormal wound repair process in COPD airways resulting in altered structure and function of airway epithelium is not established.

To test the hypothesis that wound repair of airway epithelium is abnormal in COPD, we studied primary bronchial and bronchiolar epithelial cells from non COPD and COPD patients in a model of wound closure. The relationships between airway epithelium wound closure parameters and clinical, functional and morphological characteristics of patients were studied.

## Methods

### Patients

Patients scheduled for lung resection for cancer (University Hospital of Reims, France) or transplantation (University Hospital of Grenoble, France) were prospectively recruited for the study following standards established and approved by the institutional review board of the university hospital of Reims, France. Informed consent was obtained from all the patients. Patients with asthma, cystic fibrosis, bronchiectasis or pulmonary fibrosis were excluded. At inclusion, the following characteristics were recorded: age, sex, body mass index (BMI), smoking history, arterial blood gases and pulmonary function tests results. No patient was treated by N-acetyl cysteine. COPD was defined by post-bronchodilator FEV_1_/FVC < 70% [2]. The severity of COPD was determined by spirometric classification (GOLD 1: FEV_1_ ≥ 80% predicted, GOLD 2: 50% ≤ FEV_1_ < 80% predicted, GOLD 3: 30% ≤ FEV_1_ < 50% predicted, GOLD 4: FEV_1_ < 30% predicted) and by the GOLD combined score (A, B, C, D) (GOLD 2014).

CT-scan were analyzed by two independent investigators (JMP, GD) using a visual emphysema quantification on resected lung from 0 to 4 as previously described [7].

### Cell culture and wound closure assay

Primary bronchial epithelial cells were extracted from resected lungs distant to the tumor as previously described [8]. Briefly, bronchi were visually identified and mechanically dissected. Dissected bronchi were transferred in RPMI medium supplemented with 20 mM HEPES (Gibco, Paisley, UK) and antibiotics (200 UI/ml penicillin, 200 Ag/ml streptomycin; Gibco) and then dissociated by 0.05% type XIV collagenase (Pronase E, Sigma Aldrich, St Louis, MO) and incubated in RPMI-HEPES overnight at 4°C. Cells were counted and seeded into flasks coated with type IV collagen (Sigma, Aldrich) with the CnT-17 proliferating medium (CELLnTEc, Bern, Switzerland). Cells were then passed into a 12-well plate coated with type IV collagen and cultured in bronchial epithelial cell growth medium (BEGM) (Lonza, Walkersville, MD).

When confluent and with a similar density, cells were used for wound closure assay by a mechanical injury using a P10 pipette tip as previously described [8]. Cells were washed with PBS and culture media was added. As feasibility experiments had shown that most of wounded areas of non-COPD patients were closed 18 hours after wounding, the closure of wound area was monitored for 18 hours using a digital camera and Axiovision software (Carl Zeiss Vision GmbH, Munchen-Hallbermoos, Germany) at the start of the experiment and each 10 minutes. The wound closure assays were performed in triplicate in separate wells. Images were used to determine wound repair, calculated as percent wound area compared with the initial wound area. We determined the mean speed of wound closure using Image J software (National Institutes of Health, Bethesda, MD, USA).

Seven samples (2 non COPD patients, 2 COPD GOLD II, 3 COPD GOLD IV) have been excluded because of cell culture failure: absence of initial cell proliferation (n = 5), cell death after injury (n = 1), fungal contamination (n = 1). We analysed cell growth in the initial culture depending on COPD status and severity. The time to confluence was: 7.28 ± 1.97 days in non COPD group, 7.73 ± 3.13 days in COPD group, 6 ± 2.82 days in COPD GOLD I, 7.5 ± 3.11 days in COPD GOLD II, 10.33 ± 2.52 days in COPD GOLD III-IV. However, this difference did not reach a significant level.

In a subset of patients, bronchiolar epithelial cells were also extracted from resected lungs. Briefly, bronchioles were identified on the basis of absence of wall cartilage and an outer diameter ≤1 mm [9], and then mechanically dissected. Bronchiolar epithelial cells were obtained from bronchioles following the same process as bronchi then cultured and used for wound closure assay as described above.

### Immunofluorescence

Bronchial and bronchiolar epithelial cells were cultured on glass coverslips coated with Purecol bovine collagen solution (3 mg/ml) diluted 1:75 in sterile water. When confluent, mechanical injury was performed on cell monolayer as described above. Cells were then fixed with methanol immediately after (T0) and 18 hours (T18) after the mechanical injury. In order to analyze the dynamics of wound closure parameters, we analysed 2 other time points at 6 (T6) and 12 hours (T12) after wounding. Fixed cells were blocked with 3% bovine serum albumin for 60 min. The coverslips were incubated with a mouse monoclonal antibody against Ki-67 (1:100) (Dako, Denmark), then with Alexa Fluor 488 coupled mouse anti-IgG (1:200) (Molecular Probes, Eugene, OR, USA). Nuclei were stained with 4′,6-diamidino-6-phenylindole (DAPI, Molecular Probes, Eugene, OR, USA). The results are presented as a percentage of Ki-67 positive proliferating cells compared with total cells.

### Gelatin zymography analysis

Supernatants of bronchial epithelial cell cultures at T6, T12 and T18 were collected, centrifugated and separated on 10% polyacrylamide SDS gel containing 0.1% gelatin. The gel was washed for 1 h at room temperature in a 2% Triton X-100 solution, transferred to a 50 mm Tris–HCl/10 mm CaCl2 (pH 7.6) buffer and incubated overnight at 37°C. The gel was stained in a 0.1% Coomassie Blue (G250)/45% methanol/10% acetic acid solution and de-stained in a 10% acetic acid/20% methanol solution. The gel was then analyzed by densitometry performed using Multigauge V2.02 (Fuji film, Stamford, CT, USA).

### Cytokine analysis

Supernatants of bronchial epithelial cell cultures at T18 were tested for cytokine detection (Interleukin (IL)-2, IL-4, IL-5, IL-6, IL-8, IL-10, IL-1β, IFN-γ, GM-CSF, TNF-α) using Human Cytokine Ten-Plex kit (Invitrogen, Carlsbad, CA) following manufacturer’s recommendations.

### Histological analysis

Part of resected lungs were fixed in formalin and embedded in paraffin before cutting into serial 5 μm section and staining with hematoxylin and eosin, then observed on microscope (x100). Bronchial epithelium was analyzed in 10 different areas in 4 to 7 bronchi per patient. The following major remodeling features were quantified: denuded basement membrane, goblet cell hyperplasia, basal cell hyperplasia and squamous metaplasia. Squamous cell metaplasia was defined as a pseudostratified multilayered epithelium consisting of polygonal cells covered by flattened layer of squamous cells and absence of ciliated cell [10]. The predominant remodeling feature was attributed to each observed area. Results were expressed as the percentage of remodeling feature per all observed areas per lung.

### Statistical analysis

The data are expressed as mean values ± standard deviation or percentages. We compared wound closure parameters (mean speed of wound closure, cell proliferation index, MMPs and cytokines levels) between groups (COPD status, chronic bronchitis) using the Student *t* test or Fisher exact test. We studied correlations between wound closure parameters and clinical and functional data (FEV_1_, FEV_1_/FVC, emphysema score, age, BMI, smoking history, dyspnea score, number of exacerbations) using the Pearson correlation test. A p-value < 0.05 was considered significant.

## Results

### Patients’ characteristics

Twenty patients were included, 13 COPD patients and 7 non COPD patients. Eighteen patients underwent lung resection for lung cancer (n = 17) or kidney cancer metastasis (n = 1). Two patients had lung transplantation for emphysema. Characteristics of patients are detailed in Table 1. Pre-operative arterial blood gases were available for 11 patients. COPD patients had a lower PaO2 level compared to non COPD patients (74 ± 11 vs 90 ± 9 mmHg respectively, p = 0.02).

**Table 1:** **Characteristics of patients**

	**Non COPD**	**COPD**	**p**
n	7	13	
Male gender	71%	85%	ns
Age, years	69 ± 10 [58–87]	66 ± 10 [52–84]	ns
BMI, kg/m^2^	33 ± 9 [26–43]	25 ± 4 [19–35]	0.04
Smoking history			
Never smokers	29%	0%	ns
Ex smokers	43%	54%	ns
Pack-years	29 ± 25 [0–60]	54 ± 22 [25–100]	ns
Symptoms			
Dyspnea ≥2 mMRC	0%	31%	ns
Chronic bronchitis	14%	31%	ns
At least one exacerbation in the last year	na	31%	
Spirometry			
FEV_1_,% of predicted value	94 ± 15 [72–113]	68 ± 29 [17–109]	0.04
FVC,% of predicted value	90 ± 13 [77–114]	89 ± 23 [54–117]	ns
FEV_1_/FVC,%	77 ± 5 [73–87]	59 ± 17 [27–69]	0.01
Spirometric GOLD 1/2/3-4	na	5/5/3	
GOLD A/B/C/D	na	5/5/0/3	
CT emphysema score for the resected lobe	0.4 + 0.8 [0–2]	1.3 + 1.7 [0–4]	ns
Histological analyses,% of epithelial surface, n = 17			
Denuded basement membrane	3.8 ± 5.0 [0–13]	5.4 ± 4.0 [0–14]	ns
Goblet cell hyperplasia	51.7 ± 23.4 [7–80]	48.6 ± 24.4 [16–82]	ns
Basal cell hyperplasia	4.2 ± 4.6 [0–14]	12.6 ± 22.2 [0–70]	ns
Squamous metaplasia	2.2 ± 1.8 [0–5]	2.1 ± 3.2 [0–9]	ns
Normal	38.0 ± 22.2 [10–87]	32.7 ± 23.0 [3–66]	ns

Data are expressed as mean ± SD or number (%).

FEV_1_: Forced Expiratory Volume in one second. FVC: Forced Vital Capacity.

A visual quantification of remodelling features was performed in 17 lungs. We found the main remodelling feature was goblet cell hyperplasia (51.7 ± 23.4 and 48.6 ± 24.4% of epithelium surface in non COPD and COPD groups respectively). Basal cell hyperplasia was found in 12.6 ± 22.2% of epithelium surface in COPD group. However, the percentage of epithelium surface with remodelling features was not statistically different between COPD and non-COPD groups in our series.

### Bronchial wound closure and associations with clinical, functional and histological characteristics

**Figure 1:**
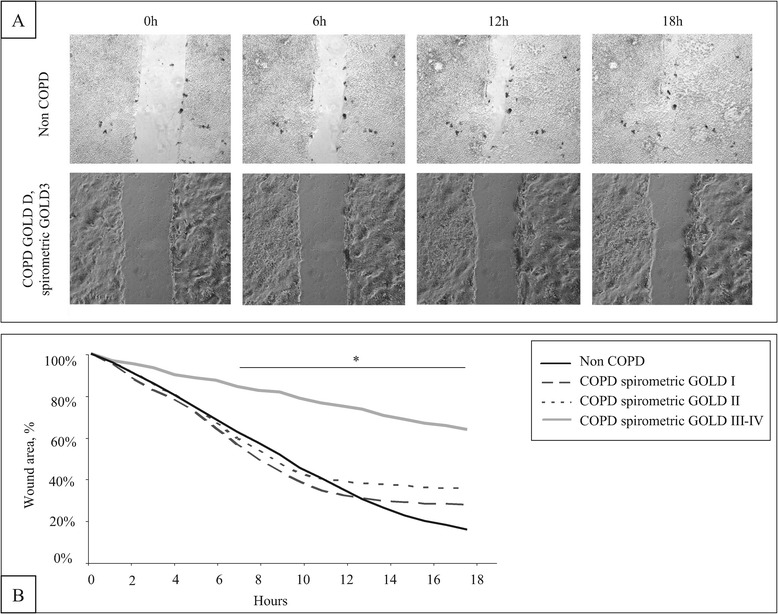
**Wound closure assay in bronchial epithelial cells from COPD and non COPD patients.** A mechanical injury was performed on confluent bronchial epithelial cell monolayer. Representative photographs at 0, 6, 12 and 18 h in a non COPD and a COPD GOLD D spirometric GOLD 3 patient **(A)**. The mean percentage of remaining wound area in non COPD patients (n = 7), spirometric COPD GOLD 1 (n = 5), GOLD 2 (n = 5) and GOLD 3–4 patients (n = 3) are presented **(B)**. *p < 0.05 vs non COPD.

**Figure 2:**
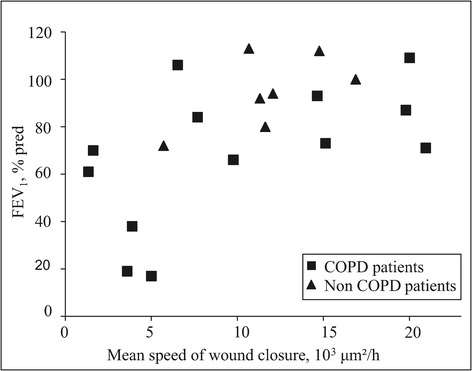
**Relationships between mean speed of bronchial epithelial wound closure and FEV**_**1**_ **.** Bronchial epithelial wound closure in non COPD (triangle, n = 7) and COPD patients (square, n = 13) was monitored for 18 h, and the mean speed of wound closure (MSWC) was calculated. p = 0.01.

To determine if bronchial wound closure is delayed in COPD, we analysed the speed of wound closure in primary bronchial epithelial cells using a wound closure assay (Figure 1A). Overall, the MSWC of COPD and non COPD patients did not significantly differ (9997 ± 7239 μm^2^/h vs 11338 ± 3552 μm^2^/h, p = 0.53). However, as shown in Figure 1B, we found that the mean speed of wound closure (MSWC) was dramatically decreased in spirometric COPD GOLD 3–4 compared to non COPD patients (4165 ± 743 μm^2^/h vs 11338 ± 3552 μm^2^/h, p = 0.006). Interestingly, the severity of airflow obstruction (FEV_1_ and FEV_1_/FVC) was associated with a lower MSWC (Table 2, Figure 2). Analysis of the subgroup of COPD patients confirmed the association between FEV_1_ and MSWC (p < 0.05). FEV_1_ was also associated with the percentage of wound area at T18 (p = 0.02, 95% CI [0.06; 0.77]). The severity of emphysema assessed by a visual score was associated with a lower MSWC (Table 2). Of notes, the MSWC was not associated with age, BMI, smoking history, dyspnea, chronic bronchitis or the number of exacerbations in the past year. Analysis of remodelling features showed that the MSWC was not associated with the percentage of denuded basement membrane (p = 0.22), goblet cell hyperplasia (p = 0.71), basal cell hyperplasia (p = 0.07), squamous metaplasia (p = 0.50) and normal epithelium (p = 0.61).

**Table 2:** **Associations between the mean speed of wound closure of bronchial epithelial cells and clinical, functional and morphological characteristics of patients**

	**P**	**95% Cl**
FEV1, % of predicted value	0.01	0.15;0.80
FEV1/FVC, %	0.04	0.03;0.76
CT emphysema score for the resected lobe	0.04	-0.77;-0.03
Age, years	0.21	-0.17;0.64
BMI, kg/m²	0.76	-0.59;0.46
Smoking history, pack-years	0.16	-0.14;0.70
Dyspnea, mMRC	0.75	-0.59;0.46
Chronic bronchitis	0.60	
Exacerbation in the past year, n	0.31	-0.67;0.26

FEV1: Forced Expiratory Volume in one second, FVC: Forced Vital Capacity.

Pearson or Student tests were performed.

### Cell proliferation during bronchial epithelial cells wound closure assay

**Figure 3:**
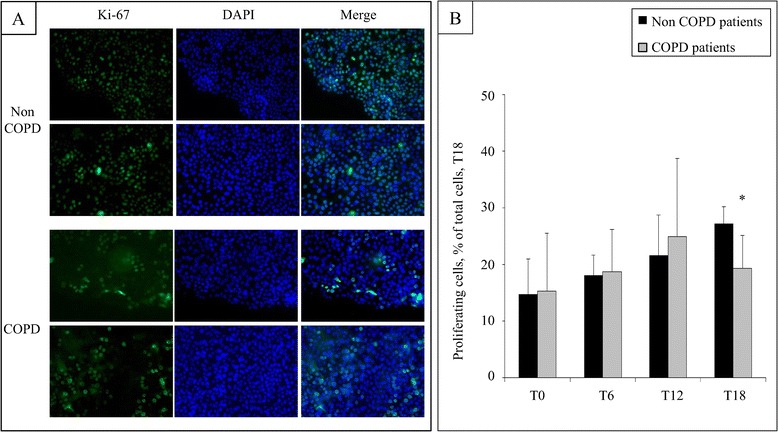
**Cell proliferation during epithelial wound closure in non COPD and COPD patients.** Representative photographs (x10 enlargement) of Ki-67 and DAPI staining, as well as merge images in non COPD and COPD bronchial epithelial cells after 18 h of repair **(A)**. Quantification of the number of Ki-67 positive cells expressed as a percentage of total cell number at 0 h, 6 h, 12 h and 18 h of repair **(B)**.

We next analysed cell proliferation during bronchial wound closure (n = 11). We performed Ki-67 and DAPI staining at T0, T6, T12 and T18 and determined the percentage of Ki-67 positive proliferating cells compared with total cells (cell proliferation index; Figure 3A). Interestingly, cell proliferation at T18 was significantly lower in COPD patients compared to non COPD patients (19 ± 6% vs 27 ± 3% respectively, p = 0.04; Figure 3B). Analysis of subgroup of COPD patients showed that cell proliferation index in COPD GOLD 3–4 patients was significantly lower compared to non COPD patients (19% ± 0.6% vs 27% ±3% respectively, p = 0.02). Cell proliferation index in COPD GOLD 1 and COPD GOLD 2 patients did not significantly differ from non COPD patients (18% ± 10% and 22% ± 6% vs 27% ± 3% respectively, ns).

We next studied associations between cell proliferation and clinical characteristics of patients. Cell proliferation index at T18 was not associated with age, BMI, smoking history, dyspnea, chronic bronchitis or the number of exacerbations in the past year (Additional file 1: Table S1). Analysis of remodelling features showed that cell proliferation index was associated with the percentage of goblet cell hyperplasia (p = 0.03, 95% CI [0.06;0.93]) but not with denuded basement membrane, basal cell hyperplasia, squamous metaplasia or normal epithelium.

Finally, we studied association between cell proliferation index and the speed of wound closure. We found that cell proliferation at T18 was not associated with the MSWC (p = 0.76). We further analysed cell proliferation at T0, T6 and T12 and did not find any association between cell proliferation index at T0, T6 and T12 and the percentage of wound closure at T6, T12 and T18 respectively.

### MMP-9 and MMP-2 levels during bronchial epithelial cells wound closure assay

We next analysed MMP-9 and MMP-2 levels in supernatants of bronchial epithelial cell cultures by gelatine zymography at T6, T12 and T18. Analysis of clinical characteristics of patients showed that MMP-9 level at T18 was negatively associated with the number of exacerbation in the past year (p = 0.04, 95% CI [−0.87;-0.04]) and positively associated with age (p = 0.02, 95% CI [0.11;0.85] but not with BMI, smoking history, dyspnea and chronic bronchitis (Additional file 2: Table S2). MMP-2 level at T18 was associated with body mass index (p < 0.01, 95% CI [0.25;0.93]) but not with age, smoking history, dyspnea, chronic bronchitis and the number of exacerbation in the past year (Additional file 2: Table S2). Interestingly, we found that MMP-2 level at T18 was significantly associated with the severity of airway obstruction (FEV_1_/FVC, p = 0.02, 95% CI [0.11;0.86] and was lower in GOLD D patients compared to GOLD A patients (7.8 ± 2 10^5^ AU vs 12.8 ± 0.13 10^5^ AU, p = 0.04). We did not find any association between MMP-9 levels at T18 and COPD status, COPD severity or emphysema (Additional file 2: Table S2). Analysis of remodelling features showed that MMP-9 and MMP-2 levels at T18 were not associated with the percentage of denuded basement membrane, goblet cell hyperplasia, basal cell hyperplasia, squamous metaplasia or normal epithelium. Levels of MMP-9 and MMP-2 in cells supernatants at T6 and T12 were not associated with clinical, functional, morphological and histological characteristics of patients (not shown).

Finally, we studied the associations between MMP-9 and MMP-2 levels and the speed of wound closure. We found that MMP-9 and MMP-2 levels at T6, T12 and T18 were not associated with the MSWC and the percentage of wound closure at T6, T12 and T18 respectively (not shown).

### Cytokine secretions during bronchial epithelial cells wound closure assay

We next analyzed pro-inflammatory cytokines levels in bronchial epithelial cell supernatants at T18. We found that the severity of airflow limitation (FEV_1_) was associated with a lower level of IL-4 (p = 0.03, 95% CI [0.09;0.87]; Additional file 3: Table S3). The levels of IL-2, IL-4, IL-10 and GM-CSF were associated with an older age of patients (p < 0.05 for all). Cytokine levels in bronchial cell supernatants at T18 were not associated with smoking history or respiratory symptoms. Analysis of remodelling features showed that IL-8 level was associated with squamous metaplasia (p = 0.01, 95% CI [0.24;0.92]). IL-2 level was associated with goblet cell hyperplasia (p = 0.03, 95% CI [0.06;0.90]).

We further studied the associations between cytokines levels and the speed of wound closure. Interestingly, higher levels of IL-4, IL-2 and GM-CSF were associated with a higher MSWC (p = 0.01, 95% CI [0.17;0.89]; p = 0.02, 95% CI [0.09;0.87]; p = 0.03, 95% CI [0.07;0.86] respectively).

### Bronchiolar epithelial cells wound closure assay

**Figure 4:**
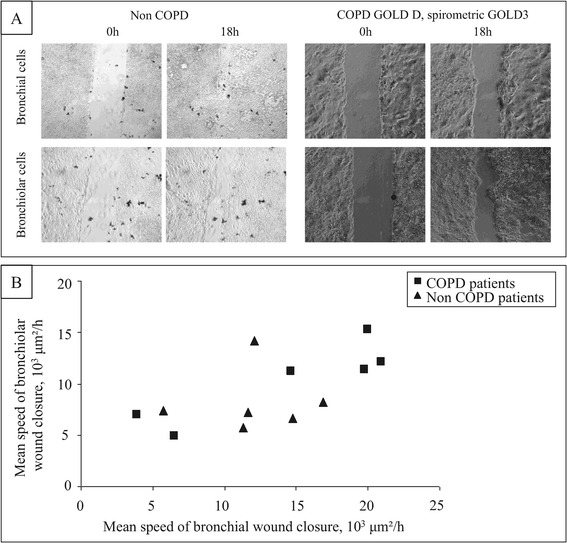
**Association between the mean speed of bronchiolar and bronchial epithelial wound closure.** Paired bronchial and bronchiolar epithelial cells were obtained in 12 patients and analysed in wound closure assay. Representative photographs at 0 and 18 h in a non COPD and a COPD GOLD D spirometric GOLD 3 patient **(A)** are presented. The association between the mean speed of wound closure of bronchiolar epithelial cells and corresponding bronchial epithelial cells is shown **(B)**. p = 0.02.

In order to determine if bronchiolar epithelial cells wound closure is similar to bronchial epithelial cells wound closure, we next analysed wound closure parameters of primary bronchiolar epithelial cells obtained from 6 non COPD patients and 6 COPD patients (4 COPD GOLD 1, 1 COPD GOLD 2, 1 COPD GOLD 3; Additional file 4: Table S4). Overall, the MSWC of bronchiolar epithelial cells was significantly lower compared to bronchial epithelial cells (9658 ± 3306 vs 13783 ± 5603 μm^2^/h respectively, p = 0.048). Analysis of paired bronchial and bronchiolar cells obtained from corresponding patient (n = 12) showed that the MSWC of bronchial and bronchiolar epithelial cells were associated (p = 0.02, 95% CI [0.12;0.89]; Figure 4). The MSWC of bronchiolar epithelial cells was not associated with COPD status or severity.

## Discussion

We showed in this study performed in primary bronchial epithelial cells obtained from 20 patients clinically, functionally and histologically characterised that the mean speed of epithelial wound closure decreased with the severity of COPD. We also showed that the speed of wound closure of bronchial and bronchiolar epithelial cells obtained from the same patients were strongly associated.

Repair of airway epithelium after injury is critical for the maintenance of the airway epithelial structure and function. In our study, we analyzed bronchial epithelium repair in the first steps of airway epithelial repair process: migration and proliferation. These processes follow the epithelial to mesenchymal transition, a dedifferentiation of epithelial cells to motile mesenchymal cells playing a key role in degradation and de novo synthesis of extracellular matrix [6]. One of the most striking findings in our study is that the delay of bronchial epithelial wound closure was associated with the severity of airflow obstruction. Moreover, we showed that COPD was associated with a decreased cell proliferation index 18 hours after bronchial epithelial cell wounding, and that cell proliferation index was not associated with the delay of bronchial epithelial cells wound closure. Several important limitations regarding the selection of the patients have to be considered in our study. First, a relatively low number of patients were included. Second, primary bronchial and bronchiolar cells were obtained mainly from lung sections distant from lung cancer. Third, the non-COPD population included ex and current smokers. However, our results strongly suggest that abnormalities in the first steps of airway epithelial repair process may be involved in severe COPD. Additional studies including a higher number of patients and using differentiated airway epithelial cells in the air liquid interface culture model would allow analyzing all different sequential steps of the repair process.

COPD is of high heterogeneity and its evaluation can not be restricted to a threshold of lung function measurement. We further analyzed parameters of clinical phenotypes of COPD patients. Interestingly, previous studies showed that airway epithelial cells repair could be impaired by senescence of airway epithelial cells or cigarette smoke exposure [11,12]. In our study, we did not find any association between MSWC or cell proliferation index and the following clinical characteristics of patients: age, BMI, smoking history, respiratory symptoms and the number of exacerbation in the previous year. Surprisingly, we did not find any statistical difference in airway remodeling features between COPD and non COPD group in our study. This could be explained by the high rate of ex or current smokers in the non COPD group, by the major heterogeneity of airway remodeling features distribution in COPD and by the relatively low number of patients analysed. In our study, goblet cell hyperplasia was associated with a higher cell proliferation index and a higher IL-2 level at T18. Squamous metaplasia was associated with a higher level of IL-8. Previous studies reported the role of cigarette smoke-mediated pro-inflammatory cytokines in the development of bronchial epithelial hyperplasia and squamous metaplasia [13]. Of notes, the MSWC was not associated with bronchial epithelial remodeling features in our study.

We analyzed MMP-2 and MMP-9 levels during wound closure assay. MMP-2 and MMP-9 have been shown to be involved in the pathogenesis of COPD [14,15]. These proteases could play a role in epithelial repair process. During epithelial repair, MMP-9 is secreted by transdifferentiated cells and degrades focal adhesion at the rear of the cell [16]. Moreover, inactivation of MMP-9 has been shown to decrease the migration of bronchial epithelial cells [17]. Previous studies showed an association between MMP-2 level and airway obstruction [18]. In our study, we found that MMP-2 level decreased in severe GOLD D COPD at T18, which may play a role in impaired bronchial epithelium repair in severe COPD. Interestingly, we found that a lower level of MMP-9 at T18 was associated with a higher frequency of exacerbation. However, we did not find any association between MMP-2 and MMP-9 levels and the MSWC in our study.

Numbers of cytokines have been shown to be involved in repair processes in the lung [19]. White and collaborators [20] found that IL-4 stimulated the migration and repair of differentiated primary human bronchial epithelial cells. IL-2 was shown to increase the migration of injured rat alveolar type II cells, and reduce apoptosis of these cells [21]. Interestingly, we found that higher levels of IL-4, IL-2 and GM-CSF were associated with a higher MSWC. Moreover, we found that IL-4 level in bronchial epithelial cells wound closure assay decreased with the severity of airway obstruction, suggesting that IL-4 could be involved in abnormal bronchial epithelial repair in COPD. Finally, a lower level of IL-4 was associated with a higher frequency of exacerbation. Further studies should explore the potential role of IL-4 as a mechanism underlying delayed repair. As previously described, we found that several cytokines were at a higher levels in older patients [22].

Finally, we analyzed bronchiolar epithelial cells in wound closure assay. Recent studies based on micro-CT imaging and histology showed a decrease in the number and the mean diameter of remodeled bronchioles in COPD [23]. A recent review suggested that bronchiolar remodeling and destruction could be involved in COPD [24]. One of the strength of our study is that we studied paired bronchial and bronchiolar epithelial cells from the same patients in a wound closure assay. We did not find any association between the MSWC of bronchiolar epithelial cells and COPD status or severity, probably because of a low number of patients and the fact that we did not obtain bronchiolar epithelial cells from the most severe patients. However, we showed that the MSWC of bronchiolar epithelial cells was strongly associated with corresponding bronchial epithelial cells.

In conclusion, our results showed that the first steps of bronchial epithelial repair are impaired in severe COPD, possibly involving MMP-2 and IL-4 and IL-10. Our results also suggest that an abnormal bronchiolar epithelium repair could be involved in COPD. Further studies are needed to determine the exact role of bronchial and bronchiolar epithelial repair after repeated injuries in the pathophysiology of COPD.

## Additional files

Additional file 1: Table S1. Associations between bronchial epithelial cell proliferation index at T18 and clinical, functional and morphological characteristics of patients. Additional file 2: Table S2. Associations between MMP-9 and MMP-2 levels in supernatants of bronchial epithelial cells at T18 and clinical, functional and morphological characteristics of patients. Additional file 3: Table S3. Associations between cytokines levels in supernatants of bronchial epithelial cells at T18 and clinical, functional and morphological characteristics of patients. Additional file 4: Table S4. Histological analyses of bronchiolar samples, n=10.

## Abbreviations

COPD: Chronic obstructive pulmonary disease
FEV_1_: Forced expiratory volume in one second
FVC: Forced vital capacity
TNF: Tumor necrosis factor
IL: Interleukin
IFN: Interferon
BMI: Body mass index
MSWC: Mean speed of wound closure
MMP: Matrix metalloproteinase
LPS: Lipopolysaccharide
