# Supplementary material for: Delay of airway epithelial wound repair in COPD is associated with airflow obstruction severity
Source: Respir Res. 2014 Nov 27;15(1):151. doi: 10.1186/s12931-014-0151-9 (PMC4251925; doi:10.1186/s12931-014-0151-9)
Supplement: Additional file 1: Table S1. — Associations between bronchial epithelial cell proliferation index at T18 and clinical, functional and morphological characteristics of patients. [file 12931_2014_151_MOESM1_ESM.doc]

Supplemental Table 1. Associations between bronchial epithelial cell proliferation index at T18 and clinical, functional and morphological characteristics of patients

|  |  |
| --- | --- |
|  | p |
| FEV1, % of predicted value | 0.41 |
| FEV1/FVC, % | 0.21 |
| CT emphysema score for the resected lobe | 0.83 |
| Age, years | 0.28 |
| BMI, kg/m^2^ | 0.76 |
| Smoking history, pack-years | 0.98 |
| Dyspnea, mMRC | 0.89 |
| Chronic bronchitis | 0.76 |
| Exacerbation in the past year, n | 0.14 |
| FEV_1_: Forced Expiratory Volume in one second , FVC: Forced Vital Capacity | |
| Spearman or Student tests were performed. |  |
