# Supplementary material for: Delay of airway epithelial wound repair in COPD is associated with airflow obstruction severity
Source: Respir Res. 2014 Nov 27;15(1):151. doi: 10.1186/s12931-014-0151-9 (PMC4251925; doi:10.1186/s12931-014-0151-9)
Supplement: Additional file 2: Table S2. — Associations between MMP-9 and MMP-2 levels in supernatants of bronchial epithelial cells at T18 and clinical, functional and morphological characteristics of patients. [file 12931_2014_151_MOESM2_ESM.doc]

Supplemental table 2. Associations between MMP-9 and MMP-2 levels in supernatants of bronchial epithelial cells at T18 and clinical, functional and morphological characteristics of patients

|  | p | |
| --- | --- | --- |
|  | MMP-9 | MMP-2 |
| FEV1, % of predicted value | 0.31 | 0.2 |
| FEV1/FVC, % | 0.3 | 0.02 |
| CT emphysema score for the resected lobe | 0.29 | 0.4 |
| Age, years | 0.02 | 0.49 |
| BMI, kg/m^2^ | 0.12 | 0.009 |
| Smoking history, pack-years | 0.45 | 0.7 |
| Dyspnea, mMRC | 0.59 | 0.73 |
| Chronic bronchitis | 0.19 | 0.35 |
| Exacerbation in the past year, n | 0.04 | 0.81 |
| FEV_1_: Forced Expiratory Volume in one second , FVC: Forced Vital Capacity | | |
| Pearson or Student tests were performed. |  |  |
