# Supplementary material for: Delay of airway epithelial wound repair in COPD is associated with airflow obstruction severity
Source: Respir Res. 2014 Nov 27;15(1):151. doi: 10.1186/s12931-014-0151-9 (PMC4251925; doi:10.1186/s12931-014-0151-9)
Supplement: Additional file 3: Table S3. — Associations between cytokines levels in supernatants of bronchial epithelial cells at T18 and clinical, functional and morphological characteristics of patients. [file 12931_2014_151_MOESM3_ESM.docx]

Supplemental table 3. Associations between cytokines levels in supernatants of bronchial epithelial cells at T18 and clinical, functional and morphological characteristics of patients

|  | IL-2 | IL-8 | IL-4 | GM-CSF | IL-5 | IL-1β | IL-10 |
| --- | --- | --- | --- | --- | --- | --- | --- |
|  |  |  |  |  |  |  |  |
| FEV_1_, % of predicted value | 0,19 | 0,94 | 0,03 | 0,32 | 0,26 | 0,17 | 0,33 |
| FEV_1_/FVC, % | 0,24 | 0,68 | 0,08 | 0,24 | 0,48 | 0,4 | 0,24 |
| CT emphysema score for the resected lobe | 0,33 | 0,45 | 0,21 | 0,19 | 0,6 | 0,47 | 0,34 |
| Age, years | 0,001 | 0,05 | 0,01 | 0,006 | 0,07 | 0,16 | 0,001 |
| BMI, kg/m² | 0,97 | 0,73 | 0,43 | 0,92 | 0,84 | 0,7 | 0,69 |
| Smoking history, pack-years | 0,47 | 0,08 | 0,82 | 0,46 | 0,3 | 0,48 | 0,2 |
| Dyspnea, mMRC | 0,71 | 0,24 | 0,38 | 0,6 | 1 | 0,72 | 0,94 |
| Chronic bronchitis | 0,42 | 0,54 | 0,11 | 0,45 | 0,39 | 0,3 | 0,28 |
| Exacerbation in the past year, n | 0,05 | 0,43 | 0,02 | 0,21 | 0,34 | 0,44 | 0,29 |
| FEV_1_: Forced Expiratory Volume in one second , FVC: Forced Vital Capacity | | |  |  |  |  |  |
| Pearson or Student tests were performed. | |  |  |  |  |  |  |
