# Supplementary material for: Delay of airway epithelial wound repair in COPD is associated with airflow obstruction severity
Source: Respir Res. 2014 Nov 27;15(1):151. doi: 10.1186/s12931-014-0151-9 (PMC4251925; doi:10.1186/s12931-014-0151-9)
Supplement: Additional file 4: Table S4. — Histological analyses of bronchiolar samples, n=10. [file 12931_2014_151_MOESM4_ESM.doc]

Supplemental table 4. Histological analyses of bronchiolar samples, n=10

|  |  | Non COPD | COPD | p |
| --- | --- | --- | --- | --- |
| Denuded basement membrane | | 0 ±0 [0–0] | 0 ±0 [0–0] | ns |
| Goblet cell hyperplasia | | 7.9 ±14.3 [0–35] | 11.6 ±13.9 [0–32] | ns |
| Basal cell hyperplasia | | 0 ±0 [0–0] | 8.3 ±16.7 [0–33] | ns |
| Squamous metaplasia |  | 0 ±0 [0–0] | 3.1 ±6.3 [0–13] | ns |
| Normal |  | 92.1 ±14 [64–100] | 75.3 ±14.3 [62–94] | ns |
| % of bronchiolar epithelial surface | |  |  |  |
| Data are expressed as mean ± SD | | | | |
